# Acne fulminans treatment: case report and literature review

**DOI:** 10.3389/fmed.2024.1450666

**Published:** 2024-07-30

**Authors:** Julia Woźna, Katarzyna Korecka, Jan Stępka, Andrzej Bałoniak, Ryszard Żaba, Robert A. Schwartz

**Affiliations:** ^1^Department of Dermatology and Venereology, Poznan University of Medical Sciences, Poznan, Poland; ^2^Dermatology, Pathology and Pediatrics, Rutgers New Jersey Medical School, Newark, NJ, United States

**Keywords:** acne fulminans, isotretinoin, acne, acne management, anti TNF-alfa therapy, corticosteorids

## Abstract

Acne fulminans (AF), a severe acne variant primarily evident in adolescent males, is characterized by the sudden onset of severe and often ulcerating acne with fever and polyarthritis. A case of a 14-year-old initially treated with clindamycin and surgical debridement, highlights the complexity of AF, including challenges in diagnosis, treatment, and the importance of early dermatological consultation. Successful management was achieved through systemic therapy with retinoids and corticosteroids, resulting in significant improvement. This case underscores the necessity of a coordinated effort among dermatologists, endocrinologists, and rheumatologists for effective AF treatment, illustrating the critical role of timely diagnosis and comprehensive care in managing this rare and challenging condition.

## Introduction

Acne fulminans (AF) represents a rare extreme manifestation of acne primarily observed in adolescent males, characterized by the abrupt onset of severe inflammatory nodular acne with systemic symptoms and abnormal laboratory findings. Typically, afflicted individuals have a history of mild to moderate acne before experiencing a sudden onset of severe, hemorrhagic, ulcerative lesions affecting the back, chest, and face, accompanied by fever, elevated white blood cell count, anemia, hepatosplenomegaly, myalgia, and arthralgia (1, 2). Roentgenographic studies may document osteolytic bone changes this condition manifests as intensely painful, hemorrhagic nodules and plaques that progress to severe necrotic ulcers, often resulting in significant scarring (3).

It is crucial to distinguish AF from acne conglobata and acne vulgaris; unlike acne vulgaris, it lacks polyporous comedones, and compared to acne conglobata, it exhibits a broader spectrum of systemic symptoms (4). Effective management of patients with this complex acne syndrome necessitates a collaborative approach involving dermatologists, endocrinologists, and rheumatologists. Additionally, AF may occur concurrently with other rare conditions such as SAPHO syndrome (synovitis, acne, pustulosis, hyperostosis, osteitis), PAPA syndrome (pyogenic arthritis, pyoderma gangrenosum, acne), PASH syndrome (pyoderma gangrenosum, acne, suppurative hidradenitis) syndrome, and CAH (congenital adrenal hyperplasia), highlighting the importance of comprehensive clinical evaluation (5).

This case report aims to provide a comprehensive overview of the clinical course, treatment strategy, and outcomes in a patient diagnosed with AF. By doing so, we aim to enhance understanding of this rare condition and emphasize the significance of timely diagnosis and multidisciplinary management.

## Case presentation

**Figure 1:**
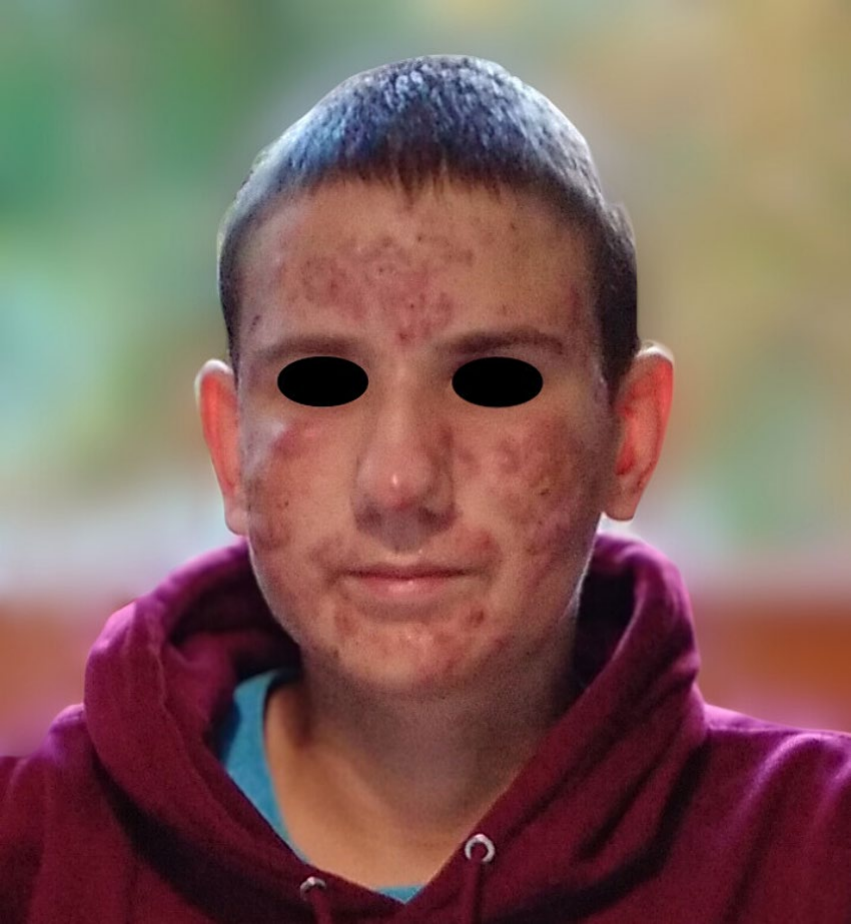
Patient’s face during treatment with tetracyclines and rupatadine, showing multiple acne lesions characterized by redness and nodules particularly on the cheeks and forehead.

A 14-year-old boy, suffering for about a year from acne (Figure 1) treated with tetracyclines and rupatadine, presented due to intensiving ankle and knee joint pain persisting of 2 weeks duration. There was no report of fever. Clinical examination revealed significant inflammatory changes on the face and milder changes on the upper back, characterized by warmth and tenderness on palpation. The worsening of acne symptoms was accompanied by an escalation in joint pain. Ultrasound examination of the facial lesions did not detect any abscesses. Sonographic assessment of the knee joints showed no signs of inflammation. However, both the right and left ankle joints exhibited swelling of the fatty tissue around the lateral malleolus, without increased fluid within the joint. Orthopedic causes of joint pain were ruled out after consultation.

Laboratory tests upon admission revealed abnormal white blood cell counts (15.42 10^9/L with 16.5% lymphocytes 1.26% monocytes, 73.9% neutrophils, 0.8% eosinophils), hemoglobin (11.7 g/dL), serum ferritin level (235.5 ng/mL), hematocrit (36.1%), creatinine (0.96 mg/dL), and fibrinogen (5.54 g/L). Elevated C-reactive protein levels were noted (69.4–17.9 mg/L).

**Figure 2:**
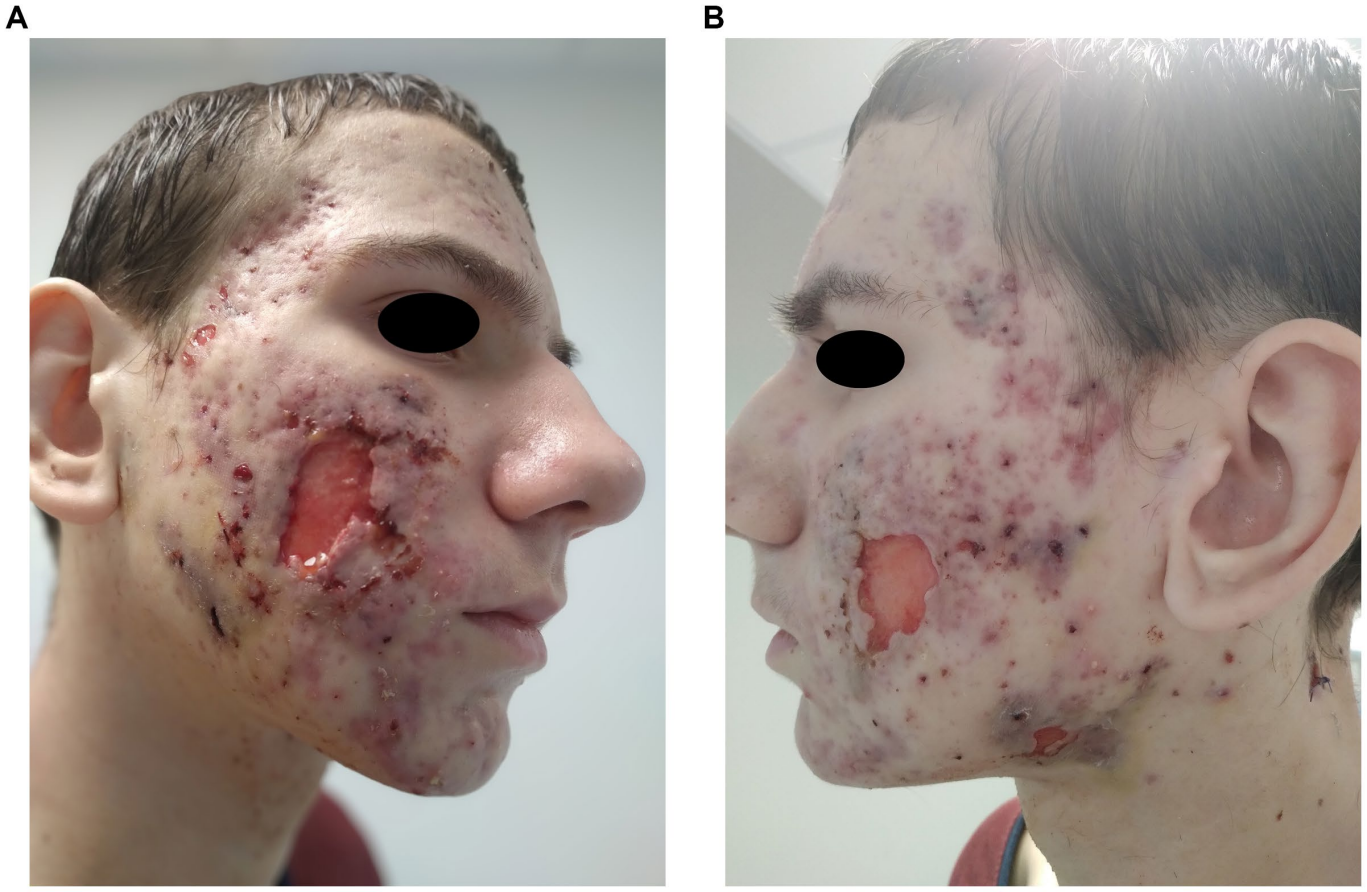
**(A,B)** Patient’s face post-surgical procedures, depicting significant skin loss on both cheeks.

During the patient’s stay in the surgery department, extensive necrosis prompted surgical debridement of the facial lesions on two occasions (due to healing complications), resulting in skin loss on both cheeks (Figure 2). *Bacteroides fragilis* was cultured from swabs of the excised lesions, and antibiotic therapy with clindamycin was initiated based on the antibiogram.

Following approximately a month of hospitalization in the surgical department, a dermatological consultation was sought, leading to the initiation of treatment with methylprednisolone 0.5 mg/kg/day for 4 weeks then tapered to 5 mg/day. After 2 weeks of methylprednisolone treatment 0.3 mg/kg/day oral isotretinoin therapy was introduced for a month, then escalated to 0.5 mg/kg/day.

**Figure 3:**
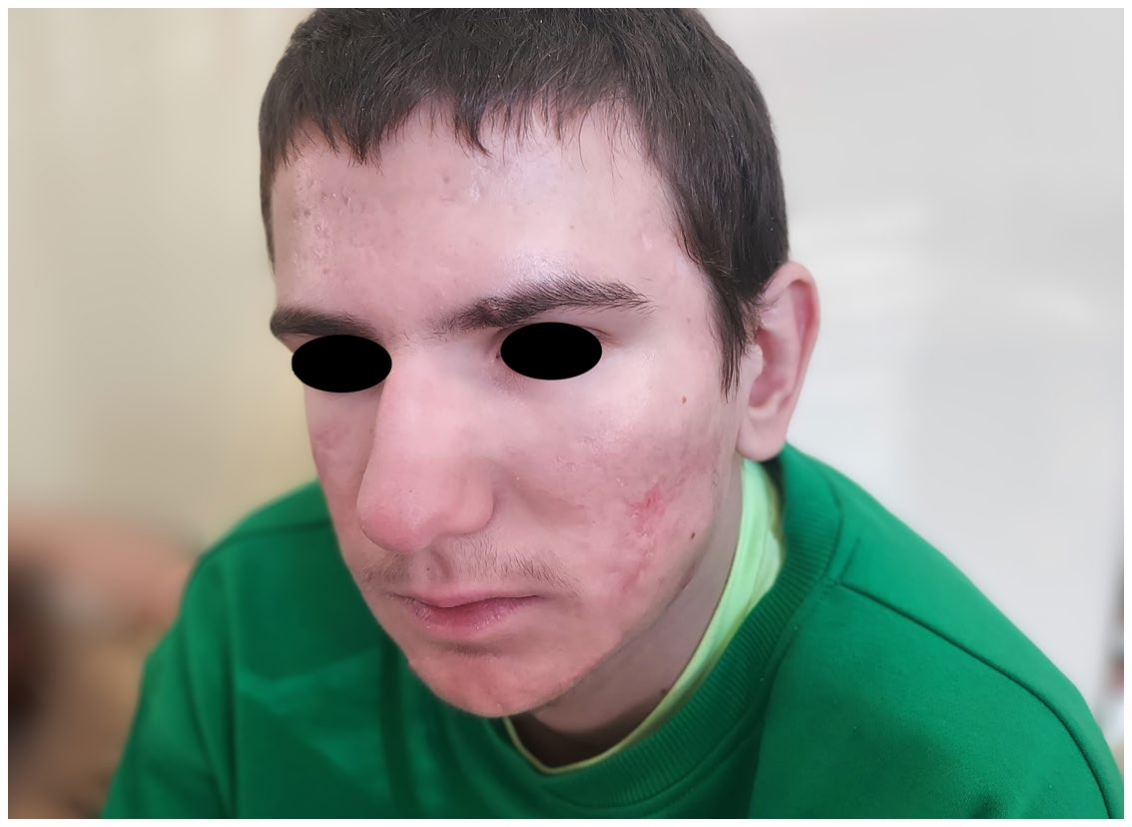
Patient’s face after treatment with isotretinoin and prednisone showing marked improvement.

A month after isotretinoine introduction, the joint symptoms resolved. After a year of isotretinoin therapy significant improvement in acne lesions was observed (Figure 3). Subsequently, the patient was referred to a nephrologist due to confirmed proteinuria during follow-up tests (exceeding 150 mg/dL) without leukocyturia, nitrituria, or hematuria, and in the absence of concurrent infection. Discontinuation of isotretinoin was considered, suspecting its impact on the occurrence of proteinuria (6). However, imaging studies showed the compression of the left renal vein between the abdominal aorta and superior mesenteric artery (nutcracker syndrome), which was deemed more likely to contribute to the proteinuria (7).

## Discussion

Etiopathogenesis of AF is is not well understood (Table 1). It is a rare condition with which many general physicians are understandably unfamiliar (16). The approach to treatment is highly individualized in this condition (Table 2).

**Table 1:** Possible causes of acne fulminans.

Category	Possible cause	Description
Infection	Cutibacterium acnes	AF may be related to hypersensitivity reaction to C. acnes antigens or chemoattractants.
	Viral Infections	Viral infections, such as measles, may trigger AF in predisposed individuals due to inflammatory cytokine release (8).
Genetic factors	Genetic Predisposition	Associated with SAPHO, PAPA, PASH, and PAPASH syndromes, indicating a genetic component (9, 10).
		Genetically determined impairment in phagocytic activity toward Cutibacterium acnes (11).
	HLA Phenotypes	Observed in monozygotic twins and siblings with identical HLA-B27 and HLAcw6 genes (4).
Immunological	Immune Complex Activation by C. acnes	C. acnes behaves as a superantigen, triggering an exaggerated antibody response; implicated in excessive granulation tissue development and increased levels of IL-1β and TNF-α (12–15).
	Systemic Inflammatory Cytokinaemia	Potential autoimmune-mediated condition; circulating immune complexes and alteration of immune response observed in some patients (12, 16).
Medications	Isotretinoin	Isotretinoin-induced fragility of pilosebaceous ducts, massive contact with C. acnes antigens, can trigger AF (4, 15).
	Others	Rare cases reported with doxycycline, erythromycin, lymecycline, oxytetracycline, methylprednisolone, and interferon-α-2a (10, 17).
Hormonal factors	Androgen Excess	Elevated testosterone levels, due to therapy or anabolic steroid use, can lead to increased sebum production and density of C. acnes population and trigger AF (10, 17).

**Table 2:** Acne fulminans treatment options – summary.

**Treatment method**	**Authors**	**Year**	**Country**	**Description**
Systemic corticosteroids (prednisolone) with isotretinoin	Greywal et al. (18)	2017	United States	American Academy of Dermatology: Evidence-based recommendations for the management of acne fulminans and its variants
	Massa et al. (19)	2017	Germany	A case series of 26 patients who resolved systemic signs and showed markedly improved skin lesions in 65% of the patients after 1 month
Broad-spectrum antibiotics with prednisolone and isotretinoin	Siadat et al. (20)	2017	Iran	Successful treatment using a combination of clindamycin, levofloxacin prednisolone, and low-dose isotretinoin. Demonstrated efficacy in microbial colonization cases.
TNF-a inhibitors: adalimumab, infliximab, golimumab, etanercept.	Taudorf et al. (21)	2024	Germany	Additional treatments included antibiotics, prednisolone, methotrexate, and isotretinoin. Improvements and remissions were observed in several patients, and one patient, unresponsive to infliximab and adalimumab, was successfully treated with combined antibiotics.
Adalimumab alone	Marasca et al. (22)	2021	Italy	Adalimumab was effective in treating AF without additional medications. Used off-label for treatment-resistant and reccurent acne.
	Kontochristopoulos et al. (23)	2021	Greece	Successful treatment of AF in a patient with coexisting hidradenitis suppurativa
	Dawoud et al. (24)	2018	Saudi Arabia	Effective for treatment-resistant AF
Adalimumab with doxycycline	Rajaii et al. (25)	2018	United States	Combined treatment of adalimumab and doxycycline showed significant improvement in AF symptoms.
Adalimumab with prednisolone and minocycline	Miguel et al. (26)	2019	Germany	This combination proved effective in severe AF cases, reducing inflammation and systemic symptoms.
Anakinra with bisphosphonates and corticosteroids	Oranges et al. (27)	2017	Italy	Anakinra, an IL-1 receptor antagonist, used with bisphosphonates and corticosteroids for severe osteoarticular symptoms in AF.
Ustekinumab	Gier et al. (28)	2023	United States	IL-12/23 inhibitor Ustekinumab was used effectively in managing AF unresponsive to corticosteroid and isotretinoin therapy.
Apremilast	Sánchez-Velázquez et al. (29)	2022	Spain	Apremilast, a selective inhibitor of phosphodiesterase 4, showed promising results in refractory isotretinoin-induced AF.
Dapsone alone	Legal (30)	2020	France	Dapsone was used successfully for unresponsive to corticosteroid and isotretinoin therapy though associated with hemolytic anemia and methemoglobinemia.
Dapsone with prednisolone	Lages et al. (31) Furukawa et al. (32)	2012 2020	Brasil Japan	The combination of dapsone and prednisolone was effective though monitoring for adverse effects is necessary.
Prednisolone alone	Proença (12)	2017	Brasil	Case series of 5 patients with AF, all had a good response to corticosteroids, but had significant scarring.
Cyclosporine with isotretinoin	Giavedoni et al. (33)	2014	Spain	Cyclosporine monotherapy showed efficacy in AF management.
Cyclosporine with prednisolone	Tago et al. (34)	2011	Japan	The combination therapy of cyclosporine and isotretinoin was effective for severe AF cases.
Methotrexate with isotretinoin	Rodríguez-Lomba et al. (35)	2016	Spain	Treatment with isotretinoin and prednisolone initially improved symptoms but relapses occurred when tapering corticosteroids. Methotrexate combined with isotretinoin provided significant improvement in AF symptoms and allowing tapering off steroid.
Photodynamic therapy with isotretinoin	Hao and Wang (36)	2020	China	Photodynamic therapy combined with isotretinoin 5-aminolevulinic acid as a photosensitizer
Photodynamic therapy with prednisone	Picone et al. (37)	2022	Italy	Photodynamic therapy combined with prednisone and methyl aminolevulinate as a photosensitizer

In this instance, the initial use of antibiotics alone and surgical debridement, while apparently necessary at the time, proved ineffective. While antibiotic monotherapy has shown limited efficacy, its effectiveness may improve when considering microbial colonization or anti-inflammatory effects (12, 31). Successful treatment of AF has been described with a combination of broad-spectrum systemic antibiotics (300 mg clindamycin thrice daily and 750 mg levofloxacin daily, based on microbial colonization results) (20). However, unlike our case, this treatment regimen also included 1 mg/kg/day of oral prednisolone at the beginning of treatment, gradually tapered and supplemented with low-dose oral isotretinoin. AF typically exhibits resistance to conventional acne antibiotics (16).

Data indicate that treatment protocols combining prednisolone and isotretinoin are effective in managing systemic symptoms and achieving acne clearance (19). Such treatment, though administered relatively late, was effective in this case. Earlier dermatological consultation could have potentially prevented surgical procedures. This case underscores the importance of a well-coordinated interprofessional team in managing AF. Urgent dermatological consultations are crucial to prevent delays, advocating for a combined treatment approach (16).

Systemic corticosteroids are frequently recommended as the initial treatment for AF to promptly alleviate inflammation (38). Treatment often involves starting with prednisone, then integrating low-dose isotretinoin, with the regimen adjusted based on the patient’s response and potential health screenings to mitigate risks (18). Evidence-based recommendations include initiating treatment with prednisone at a dosage of 0.5–1 mg/kg/day as a single intervention for a minimum of 4 weeks or until crusted lesions are resolved. Subsequently, low-dose isotretinoin at 0.1 mg/kg/day may be added to the regimen. Treatment with the corticosteroid should be continued alongside the low-dose isotretinoin for at least an additional 4 weeks. Post this period, the isotretinoin dosage may be incrementally increased while the corticosteroid is gradually tapered off (18).

Although this combination appears to be effective in treating AF, various hypotheses exist regarding isotretinoin’s role in its induction. One posits that AF originates from a severe immune response (type III and IV hypersensitivity) to antigens of *Propionibacterium acnes* (*P. acnes*), possibly initiated by the weakening of the skin’s pilosebaceous ducts due to isotretinoin treatment (39). In combination therapy, isotretinoin targets the pilosebaceous unit, while prednisolone mitigates inflammation and systemic symptoms (40). Another theory proposes that a genetic predisposition to neutrophil hyperactivity leads to ineffective phagocytosis of *P. acnes*, causing mediator release that could explain the initial worsening of acne seen with isotretinoin treatment (41). However, findings from a 2024 multicenter study indicate no significant difference between isotretinoin-associated acute acne flares (IAF) and non-associated flares (NAF), challenging the notion of isotretinoin-induced acne flares (9). This study reassures physicians about prescribing oral isotretinoin for severe acne, noting that flares can occur with any acne treatment. More research is needed on isotretinoin’s role in AF pathogenesis. It is important to consider a cutaneous drug reaction when worsening acne occurs during therapy. Blood tests should always be performed, focusing on peripheral eosinophilia, which is a key parameter in evaluating severe cutaneous adverse drug reactions (42). However, in our case, the patient’s eosinophil count was slightly lowered upon admission and normalized by the second day, suggesting that an adverse drug reaction was unlikely.

Tetracyclines are not recommended as the primary option due to their limited efficacy (38). The effectiveness of combining tetracyclines with corticosteroids remains uncertain, and such antibiotics are advised only if patients cannot tolerate isotretinoin or corticosteroids, with specified dosages (43). The concurrent use of isotretinoin and tetracyclines may lead to an increased risk of pseudotumor cerebri syndrome (44).

AF represents a unique and aggressive form of inflammatory acne with elevated inflammatory markers like TNF-alpha levels (25). The intense inflammatory response seen in AF and the effectiveness of TNF-alpha inhibitors in treating similar chronic inflammatory skin conditions, including plaque psoriasis and hidradenitis suppurativa, have led to the off-label use of these medications by dermatologists for treatment-resistant acne (45, 46). Treating AF poses a significant challenge as conventional treatments often fall short. As mentioned, the standard approach includes a regimen of oral steroids and isotretinoin. However, for cases that do not respond to these methods, alternative treatments such as biologic medications may offer relief (18, 21). TNF-alpha inhibitors have proven to be particularly beneficial in the management of AF that does not respond to prior treatments (21, 22, 25, 26). They are also effective in cases of AF with concurrent conditions such as mentioned hidradenitis suppurativa (23), SAPHO syndrome (47), and complications arising from isotretinoin therapy, such as acute sacroiliitis (24). Additionally, TNF-alpha inhibitors can reduce the need for prolonged prednisolone use, which is particularly advantageous in pediatric patients to mitigate risks like reduced growth rates and short stature (26). Other successful treatments have been documented. They include using subcutaneous adalimumab in combination with oral doxycycline (25), adalimumab with prednisolone and minocycline (26), adalimumab alone (22, 23), and infliximab paired with methotrexate (47). Moreover, alternative biologic therapies have been explored for AF treatment, including anakinra (an IL-1 receptor antagonist) used alongside bisphosphonates and corticosteroids for AF with severe osteoarticular symptoms (27), ustekinumab (an interleukin (IL)-12/23 inhibitor) (28), and apremilast (a selective inhibitor of phosphodiesterase 4) (29).

Several cases have demonstrated success in treating AF through the use of dapsone (30, 32, 48). However, treatment with dapsone is associated with two significant adverse effects: hemolytic anemia and methemoglobinemia (49). In addition, cyclosporine (33, 34) and a combination therapy of methotrexate with isotretinoin (35) have been identified as viable alternative treatment options in instances where conventional therapies are ineffective.

Effective treatment was also described with photodynamic therapy and isotretinoin with methyl aminolevulinate (37) and 5-aminolevulinic acid (ALA-PDT) used as a photosensitizer (38).Combining ALA-PDT with oral isotretinoin has been shown to clear acne lesions significantly faster and more efficiently compared to using isotretinoin alone, while also utilizing a lower dose of isotretinoin than is typically used in treatment.

## Conclusion

This case highlights the complexities in managing acne fulminans and the importance of a multidisciplinary approach for effective treatment. Timely diagnosis and appropriate interventions, including systemic therapy with retinoids and corticosteroids, significantly improved the patient’s condition.

## Data availability statement

The raw data supporting the conclusions of this article will be made available by the authors, without undue reservation.

## Ethics statement

Written informed consent was obtained from the individual(s), and minor(s)’ legal guardian/next of kin, for the publication of any potentially identifiable images or data included in this article.

## Author contributions

JW: Conceptualization, Data curation, Project administration, Writing – original draft. KK: Data curation, Supervision, Writing – review & editing. JS: Writing – original draft. AB: Writing – original draft. RŻ: Conceptualization, Data curation, Supervision, Writing – review & editing. RS: Supervision, Writing – review & editing.
